# Barcoded Sleeping Beauty Transposons as Mobile Reporters of Chromatin Architecture

**DOI:** 10.3390/ijms27188348

**Published:** 2026-09-19

**Authors:** Anna Khabarova, Miroslav Nuriddinov, Alexander Smirnov, Nariman Battulin

**Affiliations:** 1 Institute of Cytology and Genetics, Siberian Branch of the Russian Academy of Sciences, 630090 Novosibirsk, Russiabattulin@bionet.nsc.ru (N.B.); 2 Department of Genetics and Life Sciences, Sirius University of Science and Technology, 354340 Sochi, Russia; 3 Department of Natural Sciences, Novosibirsk State University, 630090 Novosibirsk, Russia

**Keywords:** Sleeping Beauty transposon, transposition, chromatin architecture, topologically associating domain, Hi-C, chromatin states, directional asymmetry, HCT116, TMUR-seq

## Abstract

Transposon integration is influenced by genomic context, but the contribution of three-dimensional genome organization to transposon mobility remains poorly understood. Here, we developed T7-Mediated Unique-barcode Recovery sequencing (TMUR-seq), combining T7-based junction enrichment with uniquely barcoded Sleeping Beauty (SB) transposons to track secondary transposition events and their relationships to individual donor sites. Using this approach, we characterized genome-wide secondary SB transposition in human cells and analyzed the effects of genomic distance, donor-specific directionality, and chromatin organization on target selection. Secondary transposition was strongly dependent on linear genomic distance but showed pronounced donor-specific directional asymmetries, including recurrent mirror-image patterns at neighboring donor sites. Secondary integratiotes also showed enrichment for Hi-C contacts with their donor loci within approximately 50 kb after accounting for genomic distance, consistent with a contribution of local three-dimensional chromatin organization to target selection. An extended chromosome 2 donor cluster further illustrated how donor distribution, chromatin state, domain organization, and local three-dimensional connectivity can coincide with highly asymmetric integration patterns and regions of pronounced integration depletion. Together, these findings indicate that secondary SB transposition is not entirely random and is shaped by the combined effects of genomic distance and local genomic context. TMUR-seq provides a framework for tracking donor–recipient relationships and investigating the genomic determinants of transposon mobility.

## 1. Introduction

Transposable elements are mobile genetic elements capable of inserting into new genomic locations, and their engineered derivatives have become indispensable tools in molecular biology and gene therapy. Among DNA transposon systems developed for use in vertebrate cells, Sleeping Beauty (SB) and piggyBac stand out for their efficient stable genomic integration, high cargo capacity, and non-viral delivery [1,2,3]. The SB transposon system, reconstructed from inactive fish transposons, integrates preferentially into TA dinucleotides distributed across the genome with a near-random profile, making it particularly attractive for unbiased functional genomics and therapeutic gene delivery [4,5]. Accordingly, SB-based vectors are currently employed in clinical trials for adoptive T-cell therapies and other gene therapy applications [6,7], where the precise genomic location of each integration event carries direct safety and efficacy implications.

The genomic integration site of a transposon is not merely a technical detail—it fundamentally determines the transcriptional fate of the integrated transgene. Decades of reporter gene studies have established that identical genetic constructs can display profound differences in expression depending solely on their chromosomal position, a phenomenon known as the position effect [8,9,10]. This variability reflects the hierarchical organization of the mammalian genome into structurally and functionally distinct chromatin domains, whose boundaries constrain regulatory activity and insulate neighboring loci from one another [11,12]. The local chromatin environment, defined by nucleosome occupancy, histone modification state, DNA accessibility, and the binding of architectural proteins such as CTCF and cohesin, determines whether a given locus is permissive or refractory to transposon integration, and shapes the transcriptional output of any transgene that lands within it [13,14,15]. Understanding how chromatin organization governs transposon behavior is therefore relevant both to the basic biology of mobile elements and to the rational design of safer gene delivery vectors.

Addressing this question requires methods capable of mapping transposon integration sites at scale and with high precision. Classical approaches—inverse PCR, ligation-mediated PCR, and splinkerette PCR—are low-throughput and prone to amplification bias [16,17]. More recent tagmentation-based methods such as TagMap and esTag-PCR improved throughput but remain limited in sensitivity in complex genomes with extensive repetitive sequences [17,18]. A conceptually distinct strategy exploits the T7 bacteriophage RNA polymerase promoter embedded within the transposon inverted terminal repeat: in vitro transcription from this promoter generates RNA anchored to the integration junction, which is then reverse-transcribed and amplified to produce a sequencing library enriched for transposon—genome junctions [19,20,21]. This T7-assisted approach, originally developed for piggyBac mapping in K562 cells21, offers a high signal-to-noise ratio and is compatible with massively parallel sequencing. A critical enhancement of this strategy is the incorporation of unique molecular barcodes into each transposon copy prior to integration, so that every insertion event carries a distinct identifier. This barcode-per-integration design enables unambiguous tracking of individual transposon copies across experiments and cell populations, and is essential for quantitative analysis of integration site distributions [21,22].

The tendency of SB to undergo local hopping—preferential re-integration near its donor site following re-mobilization—provides a unique opportunity to investigate how local genome organization influences transposition. By introducing a library of uniquely barcoded transposons, mapping their primary integration sites, and subsequently re-activating transposition, the fate of each donor insertion can be followed independently. Because every secondary integration event originates from a known genomic position, the resulting patterns of re-integration can be compared with local chromatin features, including accessibility, chromatin architecture, and three-dimensional genome organization [23,24]. Rather than serving solely as a mutagenesis tool, barcoded transposons therefore have the potential to act as mobile genomic reporters of the chromatin environment.

Here, we applied this strategy to SB transposition in HCT116 cells using uniquely barcoded transposons combined with T7-based integration site mapping—TMUR-seq. The unique barcodes enabled unambiguous identification of individual donor integration sites and tracking of their corresponding secondary transposition events following transposase re-expression. By comparing primary and secondary integration landscapes, we characterized the genomic distribution of secondary transposition events, identified reproducible donor-specific directional asymmetries, and explored whether patterns of secondary transposition are associated with local chromatin architecture and three-dimensional chromatin contacts. Together, our results show that although secondary SB transposition is governed primarily by linear genomic distance, local chromatin contacts and genomic context may further bias target selection within the immediate vicinity of the donor locus, demonstrating the potential of barcoded transposons as mobile genomic reporters for studying genome organization in living cells.

## 2. Results

### 2.1. Generation and Characterization of HCT116 Subclones Carrying Mapped SB Insertions

**Figure 1:**
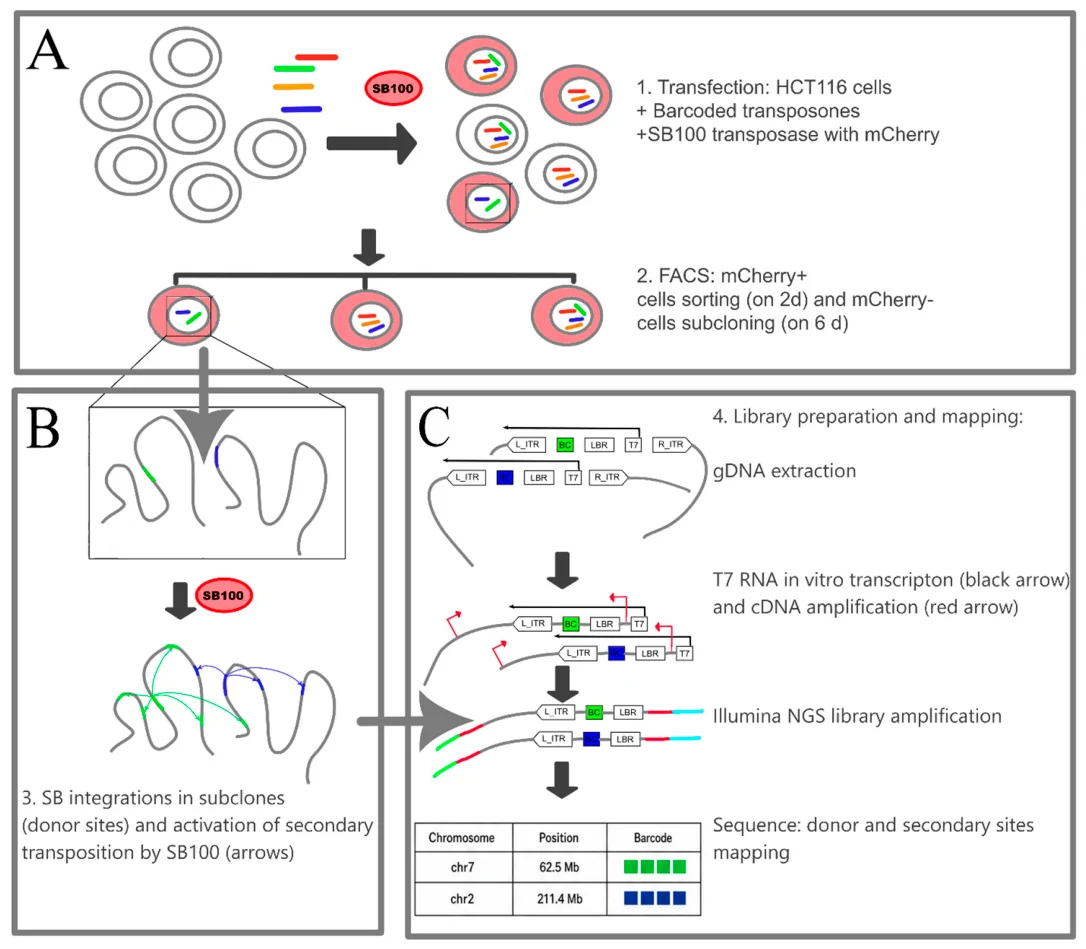
Experimental design for tracking donor and secondary SB transposition using TMUR-seq. (**A**) Experimental design. HCT116 cells were transfected with a library of uniquely barcoded Sleeping Beauty transposons (shown as colored segments) together with SB100X transposase (visualized as red fluorescence in the cytoplasm). mCherry-positive cells were isolated by FACS (Fluorescence-Activated Cell Sorting) and expanded as single-cell subclones. (**B**) Secondary transposition. Following re-expression of SB100X transposase, barcoded donor transposons generated secondary integration events, each retaining the original barcode and enabling donor–recipient relationships to be reconstructed. The two donor integration sites are shown in blue and green, with secondary integration events indicated by blue and green arrows, respectively. (**C**) Mapping strategy. Genomic DNA was processed using T7 promoter-driven in vitro transcription (black arrows) followed by cDNA synthesis (red arrows and segments) and Illumina library preparation (light blue and light green segments). Sequencing identified both donor and secondary integration sites together with their unique transposon barcodes (blue and green blocks in the table).

To generate cell lines with defined, genomically mapped SB transposon insertions, HCT116 cells were transfected with an SB transposon construct carrying a unique 16 bp molecular barcode and an SB100X transposase with an mCherry plasmid. mCherry-positive cells were enriched by FACS 24 h post-transfection to select for successful transposition events (Figure 1A). To minimize capture of secondary transposition events, cells were maintained for an additional six days to allow transposase clearance, after which mCherry-negative single cells were sorted into 96-well plates to establish clonal lines. Of 24 successfully expanded subclones, all harbored confirmed genomic SB insertion. Three subclones (SC7, SC13, and SC15) were selected for further analysis (Figure 1A). For each subclone, integration sites were mapped, and each insertion event was assigned a unique barcode, allowing unambiguous tracking of individual integrations (Figure 1B,C).

Using TMUR-seq, we mapped transposon integration sites across three HCT116 subclones. In total, 101 integration sites were identified across the three subclones: 50 in SC7, 33 in SC13, and 18 in SC15 (Supplementary Table S1). Molecular barcodes did not uniquely correspond to individual integration sites in all cases. In SC7, 38 of 50 integration sites were associated with unique barcodes, whereas the remaining 12 sites comprised six pairs of integration sites sharing the same barcode. In SC13, 16 integration sites were associated with unique barcodes, while 17 integration sites were represented by six barcodes occurring at multiple genomic locations.

A minimum read-depth threshold was applied together with the reproducibility filter to enrich the final dataset for high-confidence integration events while reducing sequencing and amplification noise. Although false-positive and false-negative rates could not be determined in the absence of orthogonal validation, the reproducible identification of the same donor integration sites across independent replicates of each subclone provides strong evidence that the detected SB insertions reflect genuine biological events rather than stochastic technical artifacts. For barcodes associated with multiple integration sites, secondary insertions generally formed distinct local clusters around one of the barcode-associated sites, allowing the predominant donor-associated distribution to be resolved. This was particularly evident for intrachromosomal events, which comprised the majority of secondary insertions. Interchromosomal secondary insertions were comparatively rare and therefore contributed little to the overall jump-distance distribution. Thus, although shared barcodes introduce some uncertainty in assigning individual secondary insertions to donor sites, this ambiguity was unlikely to substantially affect the overall jump-distance analysis.

### 2.2. Integration Sites

**Figure 2:**
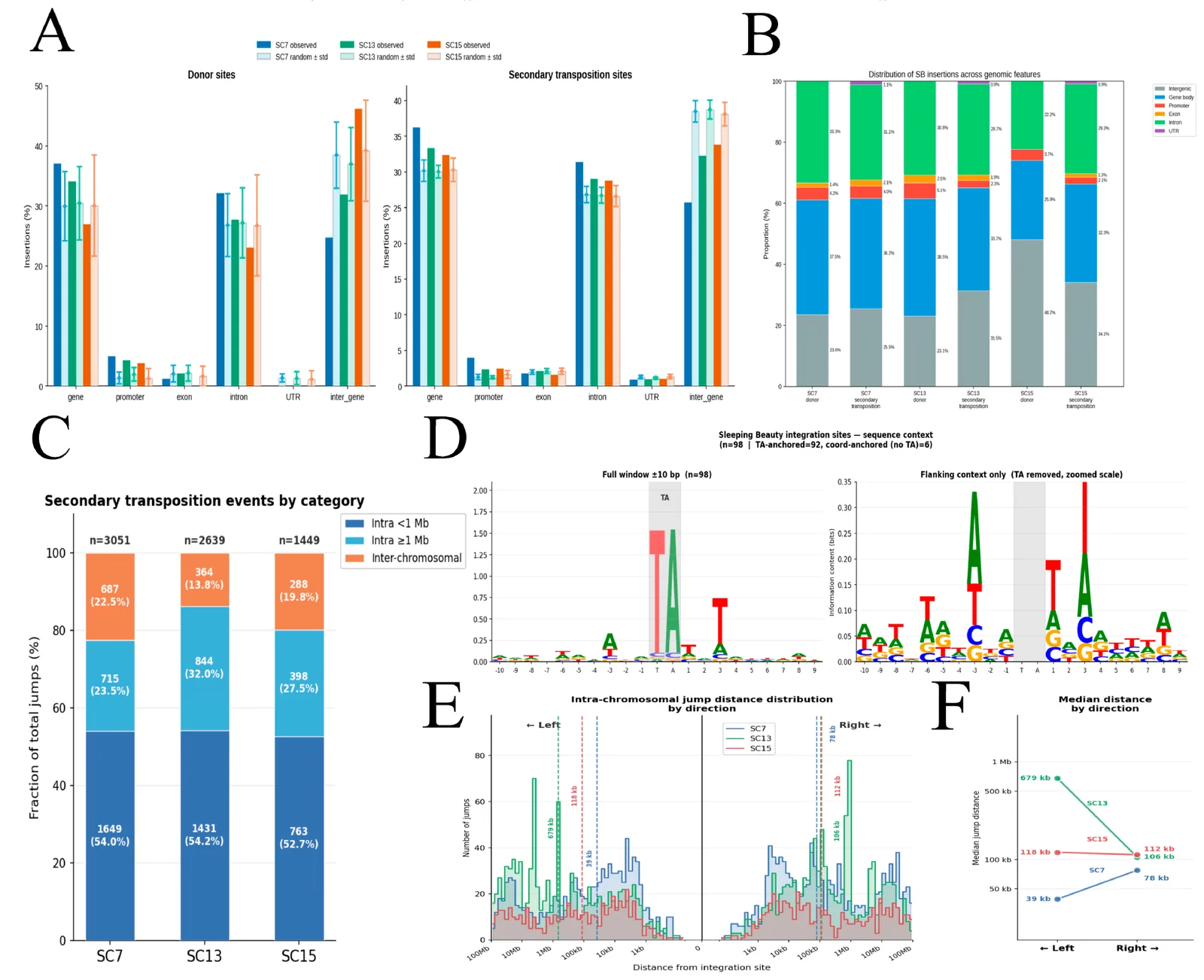
Genomic characteristics of donor and secondary Sleeping Beauty integration sites. (**A**,**B**) Genomic annotation of primary donor integration sites and secondary integration sites. Bars show the percentage of integrations within major genomic features for each subclone. (**C**) Distribution of secondary transposition events by genomic category. Stacked bars show the fractions of intra-chromosomal (<1 Mb and >1 Mb), inter-chromosomal, and donor-site events for each subclone. (**D**) Nucleotide sequence context of SB integration sites. Left, sequence logo centered on the integration site (±10 bp). Right, flanking sequence logo after removal of the conserved TA target dinucleotide, highlighting weak nucleotide preferences outside the integration site. (**E**) Distribution of intra-chromosomal jump distances to the left and right of donor sites for each subclone. Dashed lines indicate the median jump distance. (**F**) Median intra-chromosomal jump distance by direction for each subclone, illustrating directional differences in secondary transposition.

Using the Ensembl GRCh38 annotation, integration sites were classified into six categories: gene body, promoter, exon, intron, UTR, and intergenic. The genomic distribution of both the primary (donor) integration sites and the secondary transposition sites (sites resulting from transposase re-expression) was broadly similar across all three subclones and did not show major deviations from the genome-wide distribution of genomic features (Figure 2A,B). Given the relatively small number of primary donor integrations, however, the representation of individual genomic features should be interpreted with caution.

Gene body integrations accounted for 37–60% of control (donor) sites and 49–59% of secondary transposition sites, compared to a random expectation of 43–45% (Figure 2A,B). The wide range observed in control sites reflects the small sample sizes (n = 19–50 sites per subclone), as evidenced by the large SD. Secondary transposition sites, derived from thousands of events, show a narrower and more consistent gene body enrichment of 49–59% across all three subclones. Intergenic regions were correspondingly underrepresented relative to random expectation in both conditions: 40–63% observed versus 55–57% expected by chance in controls, and 42–51% versus 55–56% in secondary sites (Figure 2A,B).

Notably, promoter regions were enriched above random in both conditions (6–8% observed vs. near 2% expected in controls; 4–7% vs. 2% in secondary sites), suggesting a mild but consistent preference for transcriptionally active chromatin. Intronic integrations drove the majority of gene body integrations (32–52% in controls, 43–51% in secondary sites), while exonic, UTR, and promoter fractions were each below 7% and broadly matched random background (Figure 2A,B). These results indicate that secondary transposition does not substantially alter the overall genomic integration landscape established by the initial SB insertion events, with both primary and secondary integrations showing a broadly similar distribution of genomic features and the previously described mild gene-body and promoter biases characteristic of SB transposition [25,26].

### 2.3. Nucleotide Context of Integration Sites

To characterize the nucleotide context of SB integration sites, sequences spanning ±10 bp around each insertion site were extracted for all 100 donor loci identified across the three subclones (SC7, SC13, and SC15), excluding two ambiguous entries. Among these, 94 sites (94%) contained the canonical TA dinucleotide at the annotated insertion coordinate, whereas the remaining 6 sites (6%) lacked a detectable TA (Figure 2C).

Sequence logo analysis revealed near-complete conservation of the central TA dinucleotide, with information content reaching 1.53 bits for T and 1.55 bits for A, close to the theoretical maximum of 2 bits (Figure 2C). Outside the TA core, sequence constraints were minimal. The strongest additional signal was observed at position +4 (IC = 0.75 bits; thymine-enriched), followed by position −3 (IC = 0.33 bits; adenine-enriched), while all other positions showed information content below 0.2 bits. Analysis of the flanking sequence independent of the central TA motif confirmed that positions +4 and −3 were the only reproducibly biased nucleotides. Together, these patterns indicate that SB integration is driven primarily by the obligatory TA dinucleotide, with only a weak preference for an AT-rich local sequence environment, consistent with previous reports of SB integration bias toward AT-rich genomic regions.

### 2.4. Jump Frequency, Distance Distribution and Directional Asymmetry of Secondary Transpositions

To characterize the post-remobilization jump distribution, we added SB transposase to each subclone and mapped new insertion sites across three independent replicates per subclone (Figure 1B,C). Jump events were identified by matching the barcode of each sequencing read to the corresponding donor integration site in the pre-transposase control cell lines, enabling precise per-cell donor assignment. Reads mapping within 200 bp of the donor site were excluded as likely residual donor-site signals rather than true jumps. We tracked barcode-tagged SB insertions across three independently derived subclones (SC7, SC13, SC15), each in 3 technical replicates (Figure 1B,C; Supplementary Table S2).

Individual secondary jump sites showed low overlap between replicates, as expected given that each cell independently selects a new insertion site and the population is sampled stochastically across replicates.

Analysis of secondary transposition events revealed a broad distance distribution spanning more than six orders of magnitude, from 200 bp to 100 Mb (Figure 2C,E). Across all three subclones, the majority of events were intra-chromosomal, with short-range insertions (<1 Mb) constituting the largest fraction: 54.0%, 54.2%, and 52.7% in SC7, SC13, and SC15, respectively. Long-range intra-chromosomal events (≥1 Mb) accounted for 23.4%, 32.0%, and 27.5% of total jumps, while inter-chromosomal transposition was least frequent at 22.5%, 13.8%, and 19.9%, respectively (Figure 2C).

The distance histograms show a broadly flat profile on a logarithmic scale without a sharp distance cutoff, indicating that SB retains the capacity for both local and genome-wide redistribution. A modest enrichment at short distances (1–30 kb) is consistent with the well-documented local hopping tendency of SB, but this enrichment is not dominant—a substantial fraction of events spans distances exceeding 1 Mb in all three subclones (Figure 2E). Analysis of directional bias revealed an asymmetry in SC13: the median jump distance to the left of the donor site was 679 kb, compared to only 106 kb to the right, a 6-fold difference (Figure 2F). This pronounced leftward bias in SC13 is driven by a cluster of donor sites on chromosome 2 with systematically elevated jump distances (see Section 2.5). In contrast, SC7 and SC15 showed no substantial directional asymmetry: median distances were 39 kb (left) vs. 78 kb (right) in SC7, and 118 kb vs. 112 kb in SC15, indicating that the directional asymmetry in SC13 reflects a donor-site-specific effect rather than a general property of the SB system (Figure 2F).

Upon re-expression of the transposase, donor transposons can be mobilized and generate new integration events at genomic locations distinct from their original donor sites. We therefore refer to these newly generated insertions as secondary integration sites. Individual donor copies may give rise to multiple independent integration events within the cell population. Because the transposase itself is not expected to have an intrinsic directional preference for either side of the donor site, the null expectation is that secondary insertions should occur symmetrically upstream and downstream of the donor site, provided that local target-site availability is comparable. Systematic deviations from this expectation could therefore reflect properties of the genomic neighborhood, such as asymmetric chromatin accessibility, domain boundaries that constrain transposase mobility in one direction, or differences in the local density of TA dinucleotides. To assess the latter possibility, we examined TA dinucleotide density upstream and downstream of asymmetric donor integration sites and found no significant difference between the two sides (Wilcoxon paired test, *p* = 0.357; Supplementary Figure S1). Thus, directional asymmetry of secondary insertions is unlikely to be explained by an asymmetric local distribution of TA dinucleotides, supporting the use of transposition directionality as a readout of local genomic organization anchored to a precisely mapped donor coordinate.

**Figure 3:**
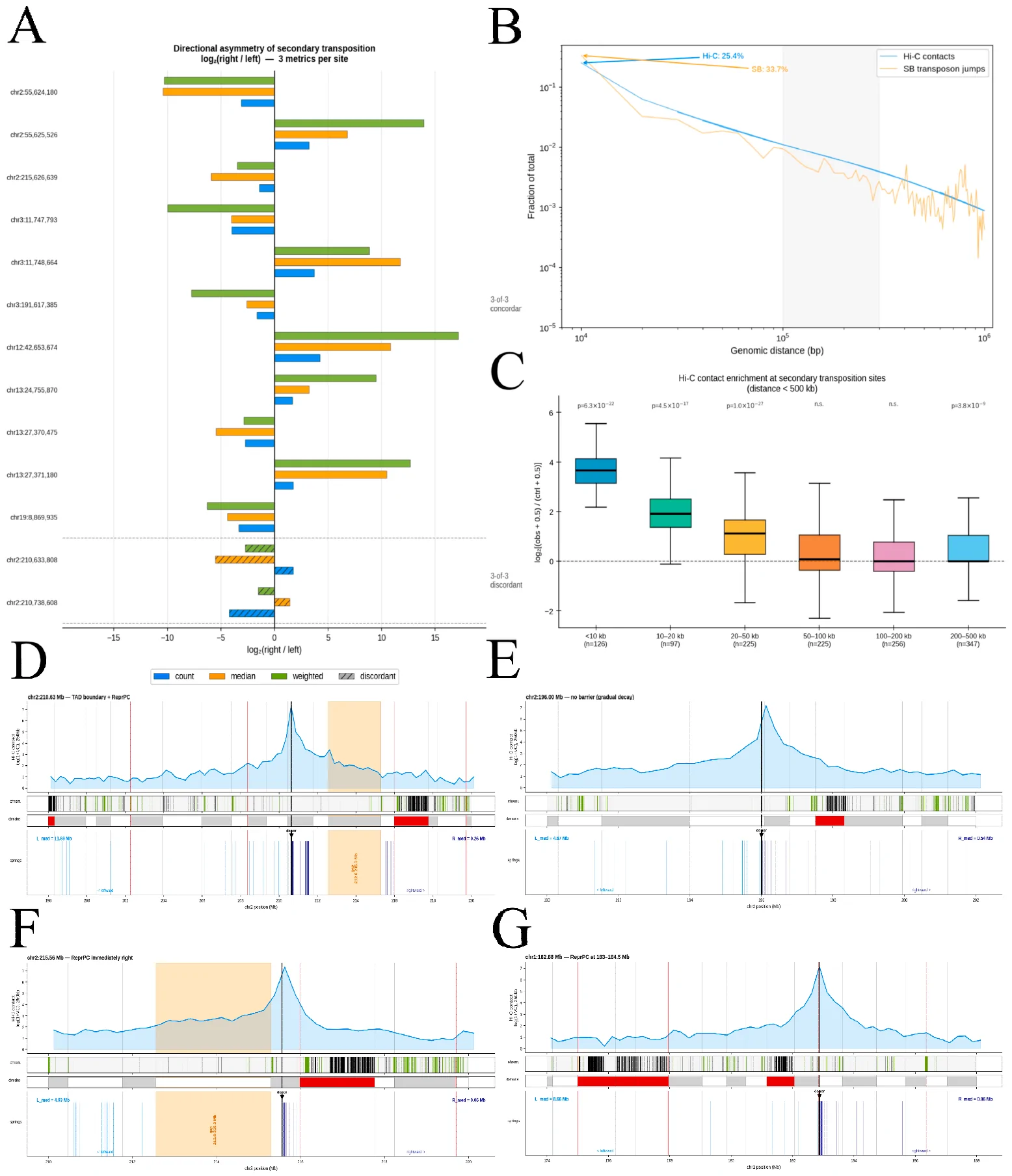
Spatial features of secondary Sleeping Beauty transposition. (**A**) Directional asymmetry of secondary transposition measured using three complementary metrics: jump count (blue), median jump distance (orange), and read-weighted mean jump distance (green). Positive log_2_(right/left) values indicate a bias toward rightward jumps and negative values toward leftward jumps. Dashed lines separate 3-of-3 concordant, discordant, and 2-of-3 concordant donor sites. (**B**) Genome-wide distance distributions of Hi-C contacts and secondary SB transposition events. Both are enriched at short genomic distances, although secondary transposition shows a steeper distance-dependent decay than Hi-C contact probability. (**C**) Hi-C contact enrichment between donor and secondary integration sites relative to distance-matched control loci. The y-axis shows log_2_[(observed + 0.5)/(control + 0.5)], where the control is the median Hi-C contact of matched loci on the same chromosome. Positive values indicate contact enrichment over the distance-dependent expectation. Boxes show genomic distance bins; the dashed line denotes no enrichment. (**D**–**G**) Asymmetric spring distributions at genomic loci with distinct chromatin and domain contexts. Four representative loci are shown: chr2:210.63 Mb, chr2:215.56 Mb, chr2:196.00 Mb, and chr1:182.88 Mb. Each locus is displayed with four tracks: (1) Hi-C 1D contact profile (4C-like, 250 kb resolution, VC normalization), with the donor site indicated by a black vertical line and TAD boundaries shown as red (strong, boundary strength ≥ 1.0) or gray (weak) lines. (2) ChromHMM chromatin states, with ReprPC shown in black, Het in dark gray, active states in green, and quiescent regions in light gray. (3) TAD domain annotation, with repressed domains (>30% ReprPC/Het coverage) shown in red and other domains shown as alternating gray and white intervals; and (4) secondary transposition springs, with leftward and rightward events shown in blue and dark blue, respectively. (L_med) and (R_med) denote the median spring distance to the left and right of the donor site, respectively. Orange shading indicates the spring-depleted region at chr2:212.6–215.3 Mb. (**D**) At chr2:210.63 Mb, springs show pronounced directional asymmetry, extending 11.69 Mb leftward but only 0.26 Mb rightward. (**E**) At chr2:196.00 Mb, no strong TAD boundary or extended repressed domain directly blocks spring propagation, and the distribution shows a more gradual decay (L_med = 4.67 Mb, R_med = 0.54 Mb). (**F**) At chr2:215.56 Mb, springs extend 4.93 Mb leftward versus 0.06 Mb rightward. The two loci flank the ~3 Mb spring-depleted region between chr2:212.6 and 215.3 Mb, which is bounded by TAD domain boundaries. (**G**) At chr1:182.88 Mb, a ReprPC-rich domain at chr1:183–184.5 Mb coincides with strong restriction of rightward spring propagation (L_med = 8.66 Mb, R_med = 0.06 Mb). Together, these loci illustrate how directional spring distributions vary with local chromatin state, TAD organization, and genomic context.

To investigate whether secondary SB transposition is constrained by local chromatin organization, we examined the directionality of transposition events relative to each donor integration site. Three complementary metrics were used to identify donor sites with reproducible leftward or rightward bias. Direct count quantified asymmetry in the number of secondary integration events occurring to the left versus the right of the donor site. Direct median compared the median genomic jump distance in each direction, capturing whether integrations preferentially occurred farther from the donor on one side than on the other. Direct weight compared the read-count-weighted mean jump distance, so that integration events supported by many sequencing reads contributed proportionally more than low-confidence events (for example, an integration supported by 100 reads contributed approximately 100-fold more than one supported by a single read). For all three metrics, directional bias was called only when the difference between leftward and rightward values exceeded two-fold. Donor sites were considered directionally concordant when at least two metrics agreed on the direction of the bias. Across the three subclones, 35 donor sites displayed directional asymmetry, including 11 sites with complete agreement across all metrics, 2 sites with discordant metrics, and 22 sites showing consistent asymmetry in jump distance but not in jump frequency (Figure 3A; Supplementary Table S3).

The 11 fully concordant sites exhibited a clear preference for transposition toward one genomic direction. Two sites showed discordance between jump-number and jump-distance metrics. Inspection of the transposition profiles revealed bimodal distributions consisting of numerous short-range insertions together with a smaller number of long-range events, consistent with heterogeneous chromatin environments in which local interactions dominate while occasional long-range contacts permit infrequent distant transpositions.

The remaining 22 donor sites showed symmetric numbers of leftward and rightward jumps but significant asymmetry in jump distance, indicating that both sides of the donor are similarly accessible for local reintegration, whereas one side provides a larger chromatin domain permissive for longer-range transposition.

To determine whether this asymmetry could instead be explained by target-site availability, we analyzed TA dinucleotide density at both local (±50 kb) and megabase (±1 Mb) scales. TA density was highly symmetric around the asymmetric donor sites and did not correlate with the preferred direction of transposition. Thus, neither local nor large-scale variation in TA abundance accounts for the observed directional bias (Supplementary Figure S1).

Taken together, these findings indicate that directional asymmetry of secondary SB transposition is primarily shaped by local chromatin architecture rather than by sequence composition. The recurrent mirror-image asymmetry observed at adjacent paired donor sites further supports the existence of nearby chromatin boundaries that redirect secondary transposition toward opposite genomic domains.

### 2.5. The chr2 Donor Cluster in SC13 Produces Pronounced Directional Asymmetry

SC13 contains seven donor sites on chromosome 2 spanning 196–216 Mb (Figure 3D–G, Supplementary Figure S2). These sites show strong directional asymmetry: at chr2:210.63 Mb (Figure 3D), the median leftward jump is 11.7 Mb versus 0.26 Mb rightward (45-fold), while at chr2:215.56 Mb (Figure 3F) the corresponding values are 4.9 and 0.06 Mb (80-fold). Similar asymmetry was observed at other donor sites across the cluster, with long-range jumps preferentially extending toward the donor-rich 196–212 Mb interval, whereas rightward jumps were largely restricted to the local donor neighborhood. Overall, chr2 donor sites produced significantly longer jumps than donor sites on other chromosomes (median 0.75 vs. 0.10 Mb). The cluster contains a ~3 Mb integration-free gap (212.5–215.5 Mb) separating two donor-rich sub-clusters: 210–212 Mb (four donors, near 800 integrations) and 215.5–216 Mb (two donors, near 200 integrations). No integrations were detected within the gap despite donors on both sides producing jumps that bypassed it (marked by orange lines in Figure 3D,F). For example, the donor at chr2:210.6 Mb (Figure 3D) generated 118 chr2 integrations, including a long-range event directly reaching the region around 215 Mb but not the 212.5–215.5 Mb zone. Conversely, donors at 215.56–215.63 Mb preferentially generated jumps toward the 210–211 Mb region (Figure 3F, orange line) or further to the right.

Interestingly, a similar pattern was observed for a donor at chr2:219.33 Mb in another subclone. This donor showed a directional asymmetry in median jump distance, with a median distance of 88.5 kb to the left versus 25.3 kb to the right. Importantly, this donor also generated an integration within the otherwise spring-free region described above (Supplementary Figure S2). Thus, the chromosome 2 gap is not completely devoid of secondary insertions across the dataset, but represents a region of pronounced and reproducible depletion that can nevertheless be bypassed or occasionally targeted by donors located outside the SC13 cluster.

Several genomic features coincide with this unusual pattern. The rightward direction from the 215.56–215.63 Mb donors is followed by a repressed chromatin (ReprPC) block at 216–218.5 Mb, defined by enrichment of H3K27me3 in the absence of active marks (H3K4me3, H3K27ac) and without H3K9me3, while the chr2:210.63 Mb donor additionally encounters a strong TAD boundary at 212.6 Mb (strength = 1.55). In contrast, the long-range leftward direction predominantly traverses quiescent chromatin toward the donor-rich region. Together, these observations indicate that the gap is associated with a combination of local domain organization and chromatin state rather than a single absolute barrier.

High donor density may provide an additional contribution. Closely spaced donor pairs occur at 215.5–215.6 Mb and 211.3–211.5 Mb. Such donor crowding could increase competition for proximal target space and make more distant integration events proportionally more prominent. We therefore consider donor density a potential contributing factor rather than evidence for direct target-site saturation.

### 2.6. Chromatin State and 3D Proximity Contribute to Target Selection

To test whether the chr2 pattern generalizes, we analyzed donor sites with the strongest directional asymmetry across chromosomes 1–4, 12, and 13. Across all tested loci, ReprPC chromatin was consistently depleted of integration events, whereas quiescent regions were preferentially targeted. Active gene-associated regions were neither significantly depleted nor enriched (Supplementary Figure S2 green blocks), indicating that transcriptional activity itself does not generally repel the transposase. Instead, the chromatin state associated with repressive marks (ReprPC) appears to be associated with reduced targeting. Genomic distance remained the dominant constraint on target selection, with 3D proximity (Hi-C contact frequency) and chromatin state providing additional modulation.

Per-site inspection revealed that the relative contribution of these factors is locus-specific. At chr2:215.56 Mb (marked by black line in Figure 3F), the block of ReprPC at 216–218.5 Mb begins less than 0.5 Mb to the right of the donor and coincides with near-complete depletion of rightward integrations: the median rightward jump is only 0.06 Mb, whereas leftward jumps reach 4.9 Mb through predominantly quiescent chromatin. A similar pattern occurs at chr1:182.88 Mb (SC7), where a ReprPC extending across 183–184.5 Mb (marked by the black line in Figure 3G) coincides with truncation of the weak direction to a median of 0.06 Mb, while the strong direction extends >8 Mb through predominantly quiescent chromatin. At chr2:210.63 Mb (marked by the black line in Figure 3D), the rightward direction encounters a strong TAD boundary at 212.6 Mb followed by the same ReprPC block; here, both domain organization and chromatin state coincide with the pronounced 45-fold asymmetry. In contrast, at chr2:196.00 Mb, the weak direction lacks a prominent RC (marked by the black line in Figure 3E) region or strong TAD boundary, and spring density declines more gradually with distance. Thus, directional asymmetry is not governed by a universal barrier, but rather reflects different combinations of distance-dependent decay, chromatin state, TAD organization, and 3D proximity whose relative contributions vary between loci.

### 2.7. Three-Dimensional Chromatin Architecture of Secondary SB Transposition

Genome-wide chromatin contacts measured by Hi-C and secondary SB transposition events both exhibited a strong preference for short genomic distances, but their distance-dependent decay differed markedly (Figure 3B). At the shortest distances, SB showed an even stronger local bias than expected from chromatin contact frequency alone: approximately one-third of all secondary integrations occurred within 20 kb of the donor site, compared with one-quarter of Hi-C contacts. Beyond ~100 kb, the two profiles diverged. Whereas the Hi-C contact probability transitioned to the characteristic shallow decay associated with TAD-scale chromatin organization, the SB distribution continued to decline approximately as a single power law without an apparent change in regime. Nearly 50% of all secondary integrations occurred within 100 kb of the donor site and 67% within 1 Mb, indicating that linear genomic distance is the primary determinant of secondary transposition. In contrast, higher-order chromatin architecture contributes little to the overall distribution of jump distances at the genome-wide scale.

To determine whether three-dimensional genome organization nevertheless influences target site selection locally, we compared the Hi-C contact frequency between each donor site and its secondary integration sites with that of distance-matched control loci (Figure 3C). Secondary SB integration sites were significantly enriched for chromatin contacts with their donor loci at genomic distances of up to 50 kb. The strongest enrichment was observed within 10 kb, where contact frequencies were approximately 13-fold higher than expected from distance-matched controls, declining to 3.8-fold and 2.2-fold in the 10–20 kb and 20–50 kb intervals, respectively. Beyond 50 kb, no consistent enrichment relative to controls was detected. Although a small number of jumps at 200–500 kb displayed elevated contact frequencies, these represented isolated events within an otherwise sparse distribution in which most donor–target pairs had no detectable Hi-C contact at 10 kb resolution. Together, these results indicate that while genomic distance largely determines the probability of secondary SB transposition, local chromatin contacts further bias integration within the immediate vicinity of the donor locus, with their influence becoming negligible beyond approximately 50 kb.

## 3. Discussion

Several methods have been developed for mapping transposon and viral integration sites, including inverse PCR, LM-PCR, LAM-PCR, tagmentation-based approaches, and whole-genome sequencing, transposon display, and Digestion-Ligation-Amplification (DLA) [27,28,29,30,31,32]. Inverse PCR, LM-PCR, and LAM-PCR rely on restriction digestion and PCR amplification of genomic sequences flanking the integration site; they are technically accessible and sensitive, but introduce strong amplification bias that can distort quantitative comparisons between sites and scale poorly to genome-wide discovery. Tagmentation-based methods, such as TagMap and esTag-PCR, replace restriction digestion with Tn5-mediated fragmentation, reducing sequence bias and improving throughput, but Tn5 itself has a preference for nucleosome-depleted regions, potentially introducing an additional accessibility bias that can confound integration frequency estimates. Whole-genome sequencing avoids these amplification and restriction-site biases but requires substantial sequencing depth to detect low-frequency events and does not readily provide per-molecule tracking.

The TMUR-seq approach used here addresses several of these limitations. First, enrichment by in vitro transcription from the junction-embedded T7 promoter is independent of restriction sites and chromatin accessibility, allowing integration sites to be recovered across diverse genomic contexts. Second, the 16 bp barcode assigned to each transposon copy before integration enables donor–recipient relationships to be tracked across secondary transposition events. Third, the combination of T7 enrichment and barcode-based filtering provides a high signal-to-noise ratio at sequencing depths compatible with routine experiments. The principal limitation of the approach is the requirement for a T7 promoter within the transposon terminal repeat, which constrains its direct application to vector systems without re-engineering. At the same time, barcoded transposons can serve not only as markers of insertion positions but also as mobile genomic reporters whose behavior can provide information about the genomic context in which transposition occurs.

An important advantage of this approach is that each secondary transposition event can be linked to a defined donor barcode, allowing large numbers of integration events to be analyzed relative to their donor loci. This provides an opportunity to study how genomic context influences transposon mobility, compare transposition behavior across genomic regions, and examine potential relationships with structural features such as chromatin domain boundaries. More broadly, the large number of secondary integration events generated in a single experiment provides a framework for studying transposition dynamics and integration preferences on a genome-wide scale.

One of the most notable observations in our study was the reproducible asymmetry of secondary transposition events. Several donor loci displayed strong directional biases, with jumps preferentially extending in one genomic direction. The origin of this effect remains unclear, but its recurrence across independent donor loci suggests that local genomic organization may influence transposon movement. Such asymmetry could reflect the combined effects of chromatin domain organization, three-dimensional chromatin contacts, local chromatin state, and the distribution of neighboring donor sites.

Our Hi-C analyses provide additional support for the contribution of three-dimensional genome organization. Although genomic distance was the dominant determinant of secondary transposition at the genome-wide scale, donor–recipient pairs within ~50 kb exhibited stronger Hi-C contacts than expected for loci at comparable linear distances. This suggests that local three-dimensional proximity may influence target selection once the strong effect of genomic distance is taken into account. Together with the recurrent directional asymmetry observed at neighboring donor sites, these findings are consistent with the contribution of local chromatin organization to secondary SB transposition. However, because the Hi-C data represent the pre-existing organization of the cell population rather than chromatin contacts measured during individual transposition events, these results should be interpreted as evidence of association rather than direct evidence of a causal mechanism.

The chromosome 2 donor cluster in SC13 provides an extreme example of this context-dependent behavior. Donors are concentrated within a ~20 Mb interval and display highly asymmetric jump distributions, with long-range events preferentially extending toward the donor-rich region. A ~3 Mb integration-free gap between two donor-rich sub-clusters shows markedly lower Hi-C connectivity with the surrounding donor-rich regions than the intra-cluster regions and is bordered by a strong TAD boundary on one side and a ReprPC region on the other. Neither feature appears to constitute an absolute barrier, as some integrations cross the boundary and occasional events from a donor in another subclone reach the gap. Moreover, expressed genes within the gap occupy only a small fraction of the region, suggesting that its pronounced depletion cannot be explained simply by gene activity. Thus, the gap likely reflects the combined effects of genomic distance, local chromatin state, domain organization, and reduced three-dimensional connectivity. The high density of nearby donor sites may provide an additional contribution by increasing competition among proximal targets, although our data do not establish a target-site saturation mechanism.

The main limitation of the approach is the requirement for substantial statistical power. Systematic analysis of chromatin-dependent effects, particularly near domain boundaries, will likely require hundreds to thousands of independent donor integrations distributed throughout the genome. Increasing library complexity also complicates barcode assignment and threshold selection, as larger numbers of integrations require proportionally deeper sequencing to distinguish true events from background noise.

A further limitation is that the barcode does not unambiguously distinguish the original donor site from secondary insertions that may have occurred before complete loss of transposase activity. Although we minimized this possibility by selecting subclones lacking detectable mCherry fluorescence, residual transposase activity may have persisted at the time of subcloning, potentially generating mosaic subclones containing multiple insertion sites associated with the same barcode. Thus, some barcode-associated integration sites may represent secondary transposition events rather than independent donor sites, potentially leading to an overestimation of the number of independent donors and affecting analyses of insertion-site preference. We therefore distinguish barcode-defined donor events from individual integration sites and interpret analyses based on the number of independent donors with appropriate caution.

A second limitation concerns the interpretation of chromatin contacts. Secondary SB transposition is strongly dominated by linear genomic distance, with nearly half of all events occurring within 100 kb of the donor locus. Consequently, distinguishing genuine effects of three-dimensional genome organization from the intrinsic local hopping behavior of the transposon remains challenging. Although comparison with distance-matched controls indicates significant enrichment of Hi-C contacts within ~50 kb, this should be regarded as evidence consistent with a contribution of local chromatin architecture rather than definitive proof of a causal mechanism. At larger genomic distances, the low frequency of secondary jumps substantially limits statistical power, making it difficult to detect more subtle chromatin-dependent effects.

In the present study, this challenge was partly addressed through subcloning, which reduced barcode complexity and enabled more reliable assignment of donor–recipient relationships. However, this came at the cost of reduced sampling density and statistical power. Despite these limitations, the method reproducibly detects non-random patterns of transposition and provides a versatile framework for studying both transposon biology and the influence of chromatin organization on genome dynamics.

## 4. Materials and Methods

### 4.1. Cloning of the Barcoded Transposon Library

This work used the Sleeping Beauty transposon system. The region between the Sleeping Beauty ITRs was 265 bp and contained, in 5′ to 3′ order, a T7 promoter (20 bp), an LBR2-derived sequence containing the Cas9 target site used in the TRIP approach [33], (85 bp), and a 16N random barcode (16 bp) (Supplementary Figure S3). The T7 promoter, which was used for transposon mapping, enabled transcription through the LBR2 sequence, the barcode, and the left ITR (462 bp total) (Supplementary Figure S3), potentially spanning over the integration junction. (R_ITR_gactcctcgctagctaatacgactcactataggggacgtgtgctcttccgatctaatttctacttcataataaagtgaactcccaggccatcgacctcttaccacttcaccatcggcaaatttcctacttggcattttcgatcacatggtcctgctggagttggtaccgatcannnnnnnnnnnnnnnnttgtggccggcccttgtgacctgcaggccttgtgactgggaaaaccctggcgtaaataaaatacgaaatgGGGCCC_L_ITR) Thus, in vitro transcription with genomic DNA generates RNA molecules containing transposon barcodes linked to integration junction sites, allowing to identify positions of transposons. The barcoded transposon library was estimated to contain at least 50,000 independent barcoded plasmid clones, as determined by counting.

*E. coli* colonies after transformation with the ligation mixture. Each colony is expected to represent an independent bacterial transformant carrying a barcoded plasmid clone. Bacterial colonies from multiple plates were pooled and used for plasmid midiprep. To facilitate transposition, SB100X transposase was expressed from the CMV-SB100X-IRES-mCherry plasmid (Supplementary Figure S3).

### 4.2. Generating Subclones with SB Integration

HCT116 cells were maintained in IMDM supplemented with 15% FBS (Thermo Fisher Scientific, Waltham, MA, USA), penicillin-streptomycin, and L-glutamine, and passaged using 0.25% trypsin. Cells were co-transfected with two constructs: an SB transposon plasmid carrying a fragment of the lamin B receptor (LBR) locus, a unique 16 bp molecular barcode, and a T7 RNA polymerase promoter; and an SB100X transposase expression plasmid fused to mCherry, at a transposase-to-transposon molar ratio of 1:4. Transfection was performed by electroporation using the Neon Transfection System (Thermo Fisher Scientific) with 300,000 cells and 2 µg total plasmid DNA (1400 V, 20 ms, 1pulse). At 24 h post-transfection, mCherry-positive cells were enriched by FACS. Cells were then cultured for an additional 6 days to allow clearance of transposase expression. At this time point, mCherry-negative single cells were sorted into 96-well plates for clonal expansion, thereby minimizing the likelihood of continued transposition during clonal expansion. No additional selection based on cellular phenotype or a specific integration pattern was applied. Expanded clones were screened to confirm the presence of Sleeping Beauty integrations, and independent clones with confirmed integrations were selected for downstream analyses. Three such clones, designated SC7, SC13, and SC15, were used in subsequent experiments. To initiate secondary transposition, SC7, SC13, and SC15 cells were transfected with the SB100X transposase plasmid alone (2 µg) under the same electroporation conditions used for the original line generation. At 48 h post-transfection, mCherry-positive cells were isolated by FACS, genomic DNA was extracted, and new transposon insertion sites were mapped.

### 4.3. NGS Library Preparation

For integration site mapping, genomic DNA was extracted from sorted cells. Barcode-containing RNA was generated by overnight in vitro transcription using the Vazyme T7 High Yield RNA Transcription Kit (TR101) according to the manufacturer’s instructions. RNA was purified using the NEB Monarch RNA Cleanup Kit, and concentration was measured prior to use; 300–1000 ng of RNA was taken for downstream processing. Reverse transcription was performed using the Vazyme HiScript III 1st Strand cDNA Synthesis Kit with gDNA Wiper (R312) according to the manufacturer’s instructions, with 0.5 µL of 100 µM RT primer p6 (5′-TCTTTCCCTACACGACGCTCTTCCGATCTNNNNNNNN-3′). cDNA was amplified using Vazyme Phanta Max Super-Fidelity DNA Polymerase (P505) with primers p7 (5′-CTGGAGTTCAGACGTGTGCTCTTCCGATCTTAATTTCTACTTCATAATAAAGTGAACT-3′) and p8 (5′-TCTTTCCCTACACGACGCTCTTCCGATCT-3′) under the following conditions: 95 °C 3 min; 16–18 cycles of 95 °C 15 s, 65 °C 15 s, 72 °C 30 s; 72 °C 1 min. PCR products were purified with 1.4× AMPure XP beads. Illumina sequencing libraries were prepared by re-amplification of 10 ng PCR product for 5–8 cycles using indexed P5/P7 primers (p5-AATGATACGGCGACCACCGAGATCTACAC(N8)ACACTCTTTCCCTACACGAC; p7-CAAGCAGAAGACGGCATACGAGATAC(N8)GACTGGAGTTCAGACGTGT). N8-index. Final libraries were purified with 1.4× AMPure XP beads and sequenced on the DNBSEQ-G400 platform in paired-end mode (2 × 150 bp).

### 4.4. Bioinformatic Data Analysis

Raw Hi-C data were obtained from the Gene Expression Omnibus (GEO) dataset GSM5983446 and re-aligned to the human reference genome GRCh38 (hg38) using juicer tools [34]. First, we filtered the libraries, excluding paired reads without LBR2 locus and ITR sequences. Then, the processed libraries were aligned by bwa (v. 0.7.18-r1243-dirty) to the hg38 human genome. The aligned read pairs were analyzed by samtools (v. 1.20) and filtered according to the following criteria:

1. Both reads in a pair must properly align;
2. One read in a pair must align on the chr1:225423850–225424080 locus (LBR2) and the second read must not align on this locus;
3. The read must have an MAPQ > 10, and one paired read must contain transposon sequence for the foregoing barcode.

For the following analysis, we realigned the processed reads.

A position of insertion was defined as the nucleotide before a transposon sequence. When the transposon sequence was not obtained, we accounted for this as the start of the alignment + 75 bp for forward-aligned reads and as the start of the alignment for reverse-aligned reads. Taking into account the error in determining the position of insertion (<10% of reads), we consolidated them within a 150 bp frame with a center in the most represented position.

Finally, we grouped the reads by barcodes. Based on the obtained data, we allowed up to 3 nucleotide changes and shifted the barcode sequence by 1 nucleotide to count reads as the same and to combine them into one group. Then, we assigned the most represented barcode to all the reads in the group. Since it is possible that differences in barcodes may originate from the initial library, we additionally considered the number of different integration sites among the compared barcodes. If differences in barcodes are due to sequencing, then such barcodes will occur at the same sites in the genome, and vice versa. Based on our observations, we empirically selected a threshold of seven unique integration sites on the genome. In cases where there were more than seven unique integration sites, these barcodes were considered different, regardless of the amount of sequence similarity. To avoid technical artifacts, we filtered every insert position–barcode combination in controls:

4. If that insert position–barcode combination is obtained in several subclones, we chose the one that was well supported by reads;
5. If several barcodes have the same insert position, we dropped it out; in that position, all barcodes supported less than 5% of reads compared to the most supported barcode;
6. The insert–barcode pair in the control must be supported by more than 3-fold reads in that insert position and by that barcode in at least one sample;
7. Around 50 kb from the insert in the sample must be obtained, with more than 6-fold reads with the barcode than in the control.

The distance of springs is defined as the genomic distance from the insertions in the samples on that chromosome with that barcode to the nearest insertion in the control on this chromosome with this barcode. If, on this chromosome in the control, the insertion with this barcode does not exist, that insertion in the samples is defined as resulting from an interchromosomal spring.

When the same molecular barcode was detected at multiple genomic integration sites, secondary insertions were assigned based on their genomic distribution rather than on the barcode alone. Specifically, secondary insertion reads were examined relative to all integration sites carrying the same barcode. In most cases, secondary insertions formed a distinct local cluster around one of the barcode-associated integration sites, allowing the corresponding donor site to be identified based on the predominant local distribution. Secondary insertions on the same chromosome were therefore assigned to the nearest barcode-associated donor site when a local cluster was evident. Closely spaced donor sites were excluded from analyses when their individual contributions could not be reliably resolved. This assignment procedure was most reliable for intrachromosomal events, which constituted the majority of secondary insertions. Interchromosomal events were comparatively rare, and their individual donor assignment could therefore be uncertain when multiple barcode-associated donor sites were present on different chromosomes. Such events were retained for analyses of genomic integration coordinates and sequence features, but this potential assignment uncertainty was considered when interpreting donor-specific jump distances.

The insertions in control are counted as asymmetric if a median spring distance in forward genomic direction differs from a median spring distance in reverse genomic direction by more than 2-fold and all counts of spring for this insertion are 10 or more.

### 4.5. Analysis of the Nucleotide Composition of SB Integration Sites

To characterize the nucleotide context of SB transposon integration sites, genomic sequences spanning ±10 bp around each donor coordinate were retrieved from the GRCh38 human reference genome. A position-specific nucleotide frequency matrix was constructed by counting the occurrence of each base (A, T, G, C) at each position from −10 to +10, where positions 0 and +1 correspond to T and A of the TA dinucleotide, respectively. Information content (IC) at each position was calculated as:

IC = 2 + b∑ fb log_2_ (fb)

where fb is the observed frequency of base b at that position, and 2 bits represents the maximum possible information content for a four-letter DNA alphabet. The resulting IC values were used to construct a sequence logo using the logomaker Python library (v0.8.7). To assess nucleotide preferences in the flanking context independently of the TA core, IC values at all non-TA positions were compared against an empirical background threshold derived from the mean IC across all flanking positions (mean = 0.11 bits).

### 4.6. Random Genome Control and TA Density Analysis at ±1 Mb

To assess whether large-scale TA dinucleotide asymmetry contributes to the directional bias of secondary SB transposition, we generated a null model consisting of 500 random genomic positions in hg38 (chromosomes 1–22 and X), restricted to sites at least 1 Mb from chromosome ends. For each site, 1 Mb upstream and downstream sequences were retrieved using the Ensembl REST API, TA dinucleotides were counted, and log_2_(TA_right/TA_left) was calculated. The same analysis was performed for all 35 asymmetric secondary transpositions. For each site, we calculated an empirical z-score relative to the null distribution and assessed concordance between TA asymmetry and observed jump direction. Directional concordance was evaluated using binomial tests, differences between LEFT- and RIGHT-biased sites by the Mann–Whitney U test, and correlation between ±50 kb and ±1 Mb TA asymmetry by Pearson correlation. To examine the spatial distribution of TA dinucleotides, 2 Mb sequences centered on each of the 33 concordant sites were analyzed using 50 kb sliding windows (25 kb step). For LEFT-direction sites, coordinates were inverted so that positive distances always corresponded to the preferred jump direction. TA densities on the preferred versus opposite sides were compared using the Wilcoxon signed-rank test.

### 4.7. Analysis of Three-Dimensional Chromatin Contacts Associated with Secondary SB Transposition

To assess whether secondary SB integration sites are enriched for three-dimensional chromatin contacts with their donor loci, we analyzed a 10 kb resolution Hi-C contact matrix from HCT116 cells (KR-normalized, hg38 assembly). For each secondary transposition event occurring on the same chromosome as its donor site (n = 3570), we extracted the observed Hi-C contact frequency between the donor and integration bins (obs_real_). As a distance-matched background, we calculated the median contact frequency across randomly selected control loci located on the same chromosome at a similar genomic distance from the donor (±5% of the donor–integration distance, maximum ±100 kb; 10–41 control loci per jump). Contact enrichment was quantified as

log_2_(obs_real_ + 0.5/median_ctr_ + 0.5)

where a pseudocount of 0.5 was added to avoid undefined ratios. A value of zero indicates no enrichment over the distance-matched background, whereas positive values indicate stronger-than-expected chromatin contacts. Statistical significance was assessed separately for six distance intervals (<10 kb, 10–20 kb, 20–50 kb, 50–100 kb, 100–200 kb, and 200–500 kb) using a one-sample Wilcoxon signed-rank test against a median of zero, with Bonferroni correction for six comparisons.

### 4.8. Comparison of Hi-C Contact Decay and SB Jump Distances

Genome-wide contact probability as a function of genomic distance (P(s)) was calculated from the KR-normalized 10 kb Hi-C map of HCT116 cells (hg38) (dataset GSM5983446) by averaging all intra-chromosomal contacts across the 22 autosomes. Secondary SB transposition distances were calculated for all intra-chromosomal donor–integration pairs (6990 events from 103 donor sites) and binned at 10 kb resolution. Both Hi-C contacts and jump frequencies were normalized as fractions of the total number of contacts or jumps, respectively, allowing direct comparison of their distance-dependent decay.

Chromatin domain boundaries were identified from the HCT116 Hi-C (dataset GSM5983446) map using an insulation score approach. The insulation score was calculated at 500 kb resolution using a sliding-window analysis of the Hi-C contact matrix. For each genomic position, the insulation score reflects the relative depletion of Hi-C contacts crossing that position, with local minima indicating potential domain boundaries.

Boundary strength was calculated as the log2 ratio of the insulation scores on the two sides of the boundary. Boundaries with a strength value > 1.0 were classified as strong boundaries, whereas boundaries with a strength ≤ 1.0 were considered weak.

### 4.9. Chromatin State Annotation

Chromatin state segmentation for HCT116 cells was obtained from ENCODE (accession ENCSR448SWW) using the ChromHMM35 15-state model on the GRCh38 assembly. ChromHMM is a multivariate hidden Markov model that identifies chromatin states based on combinatorial patterns of histone modifications [35]. The 15-state segmentation was used to annotate the local chromatin environment of donor and target regions.

For the bin-level analysis, the genome was divided into 50 kb bins, and the dominant ChromHMM state was assigned to each bin based on the largest total overlap (in bp) between the bin and the underlying ChromHMM states. The 15 states [36] were grouped into four functional categories: active (Tss, TssFlnk, TssFlnkU, TssFlnkD, Tx, Enh1, EnhG1, EnhG2), weak (TxWk, Enh2), repressed (ReprPC, Biv, Het, ZNF/Rpts), and quiescent (Quies).

Because Polycomb-repressed chromatin was of particular interest in the analysis, the ReprPC state was also considered separately from the broader repressed category. ReprPC corresponds to regions characterized predominantly by H3K27me3 enrichment and lacking the active chromatin features characteristic of promoter- and enhancer-associated states. Thus, bins whose dominant ChromHMM state was ReprPC were classified as Polycomb-repressed chromatin for the corresponding analysis.

## Data Availability

The raw sequencing data and processed files have been deposited in the NCBI site PRJNA1522909. Raw Hi-C data were obtained from the Gene Expression Omnibus (GEO) dataset GSM5983446 and re-aligned to the human reference genome GRCh38 (hg38) using juicer tools. ChromHMM segmentation data were obtained from ENCODE (accession ENCSR448SWW).

## Supplementary Materials

The following supporting information can be downloaded at https://www.mdpi.com/article/10.3390/ijms27188348/s1.

## Abbreviations

The following abbreviations are used in this manuscript:

SB	Sleeping Beauty
TMUR-seq	T7-Mediated Unique-barcode Recovery sequencing
TAD	Topologically Associating Domain
Hi-C	High-throughput Chromosome Conformation Capture
ChromHMM	Chromatin Hidden Markov Model
ReprPC	Polycomb-Repressed Chromatin
Het	Heterochromatin
VC	Vanilla Coverage normalization
KR	Knight-Ruiz normalization
FACS	Fluorescence-Activated Cell Sorting
NGS	Next-Generation Sequencing
GEO	Gene Expression Omnibus
ENCODE	Encyclopedia of DNA Elements
