# Supplementary material for: Barcoded Sleeping Beauty Transposons as Mobile Reporters of Chromatin Architecture
Source: Int J Mol Sci. 2026 Sep 19;27(18):8348. doi: 10.3390/ijms27188348 (PMC13607698; doi:10.3390/ijms27188348)
Supplement: Supplementary file 1 [file ijms-27-08348-s001.zip › Supplementary materials.pdf]

Supplementary materials

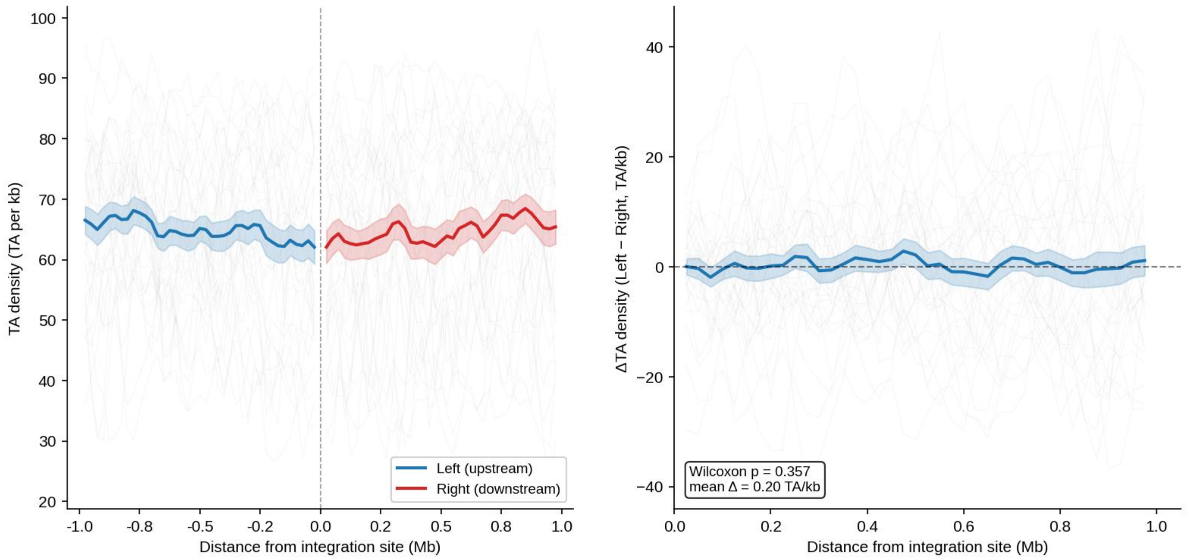

Supplementary Figure S1 Distribution of TA dinucleotides around asymmetric donor integration sites. (A) TA dinucleotide density as a function of distance from the donor integration site. Each gray line represents an individual asymmetric donor site; blue and red lines show the mean TA density upstream and downstream of the integration site, respectively. The dashed vertical line indicates the donor integration coordinate. (B) Difference in TA dinucleotide density between the upstream and downstream regions at matched distances from the donor integration site. The blue line represents the mean difference, with individual donor profiles shown in gray. The dashed horizontal line indicates zero difference. No significant asymmetry in TA density was detected between the two sides (Wilcoxon paired test, $P = 0.357$; mean difference = 0.20 TA/kb).

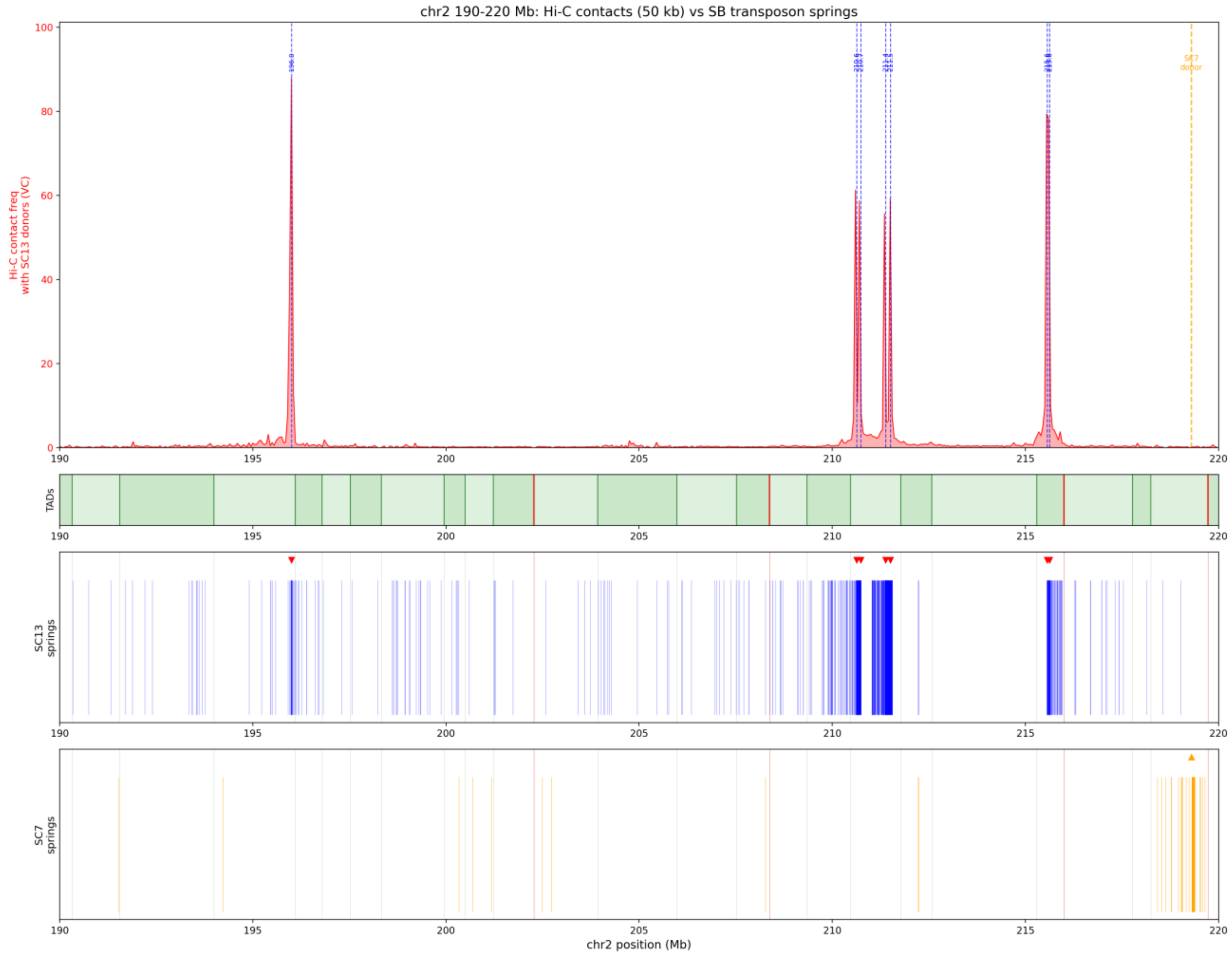

Supplementary Figure S2. Chromatin landscape of chr2:190–220 Mb and SB transposon spring distribution. Five tracks are shown from top to bottom: (1) Hi-C contact frequency between genomic bins and the SC13 donor sites at 50-kb resolution, shown as VC-normalized observed/expected contact frequency; blue dashed lines indicate SC13 donor sites, and the orange dashed line indicates the SC7 donor site at chr2:219.3 Mb; (2) TAD domain map at 500-kb resolution; alternating green shading indicates intervals between TAD boundaries, red lines indicate strong boundaries (boundary strength ≥1.0), and dark green lines indicate weak boundaries; (3) ChromHMM 15-state chromatin annotation; black indicates Polycomb-repressed chromatin (ReprPC), dark gray heterochromatin (Het), green active states (Tss/Tx/Enh), and light gray quiescent chromatin; (4) SC13 secondary transposition sites shown as a blue rug plot; (5) SC7 secondary transposition sites shown as an orange rug plot. The ~3-Mb spring-depleted region between ~212.6 and 215.3 Mb is flanked by TAD boundaries and shows reduced Hi-C connectivity with the donor-rich regions.

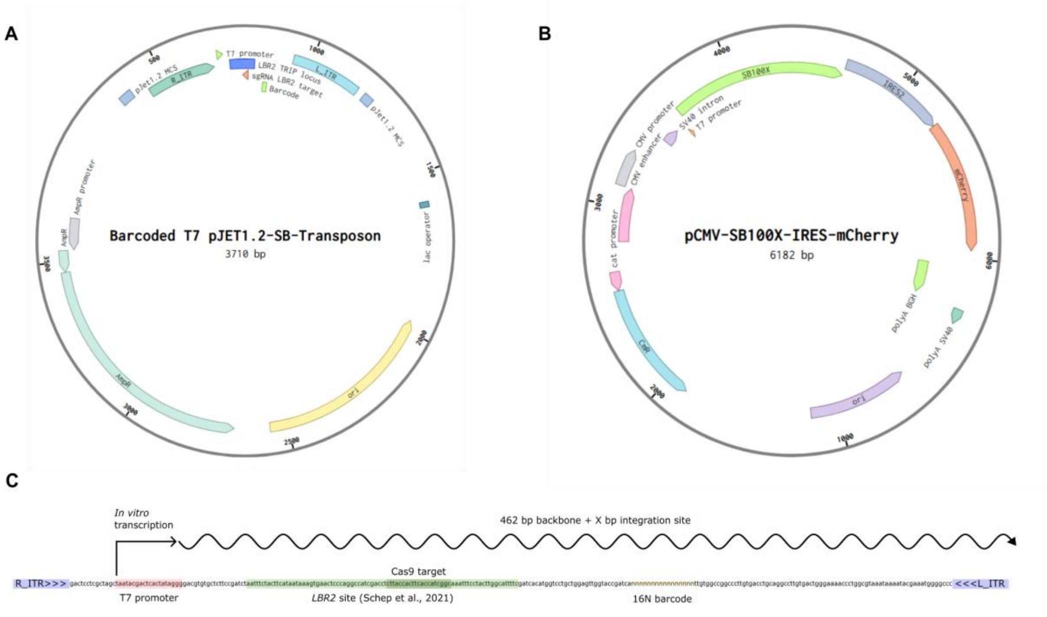

Supplementary Figure S3. Plasmid maps and sequence organization of the vectors used in the study. **(A)** Map of the barcoded Sleeping Beauty (SB) transposon assembled in the pJET1.2 backbone. The transposon contains a T7 promoter, LBR2 target site, and a 16-bp molecular barcode flanked by SB inverted terminal repeats (ITRs). **(B)** Map of the pCMV-SB100X-IRES-mCherry plasmid encoding the SB100X transposase and mCherry fluorescent marker. **(C)** Schematic of the SB transposon sequence flanked by the left and right ITRs. The positions of the T7 promoter, LBR2 site, and 16-bp barcode are indicated. The transcript generated by in vitro transcription contains a 462-bp transposon backbone upstream of the genomic integration site.
